# Mycobiome changes in the vitreous of post fever retinitis patients

**DOI:** 10.1371/journal.pone.0242138

**Published:** 2020-11-19

**Authors:** Kotakonda Arunasri, Malleswarapu Mahesh, Gumpili Sai Prashanthi, Rajagopalaboopathi Jayasudha, Sama Kalyana Chakravarthy, Mudit Tyagi, Rajeev R. Pappuru, Sisinthy Shivaji

**Affiliations:** 1 Jhaveri Microbiology Centre, Prof Brien Holden Eye Research Centre, L V Prasad Eye Institute, L V Prasad Marg, Banjara Hills, Hyderabad, India; 2 Smt. Kanuri Santhamma Centre for Vitreo Retinal Diseases, L V Prasad Eye Institute, L V Prasad Marg, Banjara Hills, Hyderabad, India; University of Hyderabad, INDIA

## Abstract

Fungi have been associated with various diseases of the eye like keratitis, uveitis and endophthalmitis. Despite this fact, fungal microbiome (mycobiome) studies compared to the bacterial microbiome studies have remained neglected. In the present study, using metagenomic sequencing, the mycobiomes of the vitreous of healthy control individuals (VC, n = 15) and individuals with post fever retinitis + non-PFR uveitis (PFR+, n = 9) were analysed and compared. The results indicated that *Ascomycota* was the most predominant phylum in both VC and PFR+ groups. Further, at the genera level it was observed that the abundance of 17 fungal genera were significantly different in post fever retinitis (PFR, n = 6) group compared to control group. Of these 17 genera, it was observed that 14 genera were relatively more abundant in PFR group and the remaining 3 genera in the VC group. Genus *Saccharomyces*, a commensal of the gut and skin, was predominantly present in the vitreous of both the cohorts, however it was significantly less abundant in PFR group. Further, significant increase in the genera that have a pathogenic interaction with the host were observed in PFR group. On the whole the mycobiome in both the groups differed significantly and formed two distinct clusters in the heatmap and Principal co-ordinate analysis. These results demonstrate significant changes in the mycobiome from the vitreous of post fever retinitis patients compared to healthy controls thus implying that dysbiotic changes in the fungal vitreous microbiome are associated with PFR.

## Introduction

Fungi are causative agents of several infections of the eye and range from infections of the ocular surface as in keratitis and could also cause intraocular infections as in endophthalmitis, uveitis and retinitis. In addition, fungi could infect eyelids, conjunctiva, and lacrimal system as in blepharitis, conjunctivitis and dacryoadenitis. Fungal infection of paranasal sinuses and other structures close to the orbit as in fungal sinusitis and acute invasive fungal rhinosinusitis was also reported [1, 2]. Among the regions of eye that the fungi infect, the ocular surface infections appear to be most prevalent. The fungi causing ocular surface infections include *Lasiodiplodia theobromae*, *Candida* spp., *Fusarium*, *Alternaria*, and *Aspergillus* [1, 3]. Several of these fungi also cause fungal endogenous endophthalmitis and include *Cryptococcus neoformans*, *Sporotrichum schenckii*, *Histoplasma capsulatum*, *Blastomyces dermatitidis*, and *Coccidioides immitis* in addition to *Aspergillus* and *Fusarium* [4]. Fugal retinitis is rare and the causative agents include *Torulopsis glabrata* [5], *Pseudomyxoma peritonei* [6] and *Candida albicans* [7].

Fungi, apart from their potential ocular pathogenicity are also associated with the healthy human eye [8, 9]. Culture-dependant methods showed that fungi could be detected on the ocular surface of healthy individuals in up to 28% of the individuals [8]. However, in a recent publication based on NGS technology fungi was identified on the ocular surface of 73.5% healthy individuals. This study also showed that genera *Aspergillus*, *Setosphaeria*, *Malassezia*, and *Haematonectria* were predominantly present in healthy eyes [9]. Interestingly all these genera are also known to be associated with ocular fungal infections [3] implying that these are opportunistic pathogens. In a subsequent study based on the abundance analysis of the fungal genera in the conjunctival swabs and corneal scrapings of fungal keratitis individuals indicated that 11 core genera namely *Aspergillus*, *Setosphaeria*, *Malassezia*, *Haematonectria*, *Candida*, *Emericella*, *Penicillium*, *Fusarium*, *Cladosporium*, *Choiromyces*, *and Cochliobolus* showed differential abundance in the keratitis patient eyes compared to healthy conjunctival swabs [10]. On a similar note in culture negative endophthalmitis patient samples, four genera *Aspergillus*, *Fusarium*, *Exserohilum* and *Candida* were observed to be predominantly present in 73.7% of the samples analysed [11]. In the present study the fungal diversity and abundance was assessed by metagenomic sequencing of the vitreous inpost fever retinitis individuals compared to the vitreous collected from individuals undergoing ocular surgery who had no ocular and systemic infection. Post fever retinitis (PFR) or retinitis post febrile illness is a form of retinitis that prevails mostly in the Indian subcontinent [12, 13]. Clinical manifestation of PFR happens after 2 to 4 weeks of post febrile illness and occurs mostly after bacterial [14], viral [15, 16] and protozoan [17] epidemics. A recent study also reported a case of fungal retinitis following a viral systemic illness in an immune competent individual [7]. Although the clinical impression of PFR and retinitis are well documented [13], changes in the intraocular microbial composition remained unknown. Thus the present study adopted next generation sequencing (NGS) approach to unravel the fungal microbiome (mycobiome) in retinitis patients compared to healthy individuals. The rationale of the present study was to ascertain whether mycobiome composition in the vitreous body was altered in PFR individuals. Our results indicated significant differences in the mycobiome in the vitreous of post PFR patients compared to healthy controls thus implying that dysbiotic changes in the fungal vitreous microbiome are associated with PFR.

## Materials and methods

### Study group and sample collection

Collection of vitreous from healthy controls would not be ethical. Therefore in this cohort we included individuals who were to undergo ocular procedures like Macular hole surgery or Rhegmatogenous retinal detachment from whom vitreous could be collected. These controls were not symptomatic for PFR and had no other ocular infections. All the participants with a past history of Typhoid, Malaria and Chikungunya and febrile illness were excluded from the study. All participants who had history of inflammatory disorders of the eye, uncontrolled glaucoma, hypertension and diabetes were excluded from the study. A total of 15 individuals were recruited as controls and 300 μl of vitreous was collected from each individual.

PFR group included individuals with PFR which normally manifested 2 to 4 weeks after systemic infections like Typhoid [14], Malaria and Chikungunya [17] caused due to a wide array of infectious agents such as bacteria, parasites and viruses [18, 19]. All individuals who presented with features of retinitis (please see introduction) and who had a past history of fever were considered to be having post fever retinitis. All patients with HIV, Tuberculosis and Syphilis were excluded from the study. Individuals with history of fever and retinitis due to Behcet's disease and systemic lupus erythematosus [20] were also eliminated. PFR individuals who had inflammatory disorders of the eye, uncontrolled glaucoma, hypertension and diabetes were also excluded from the study. In both control and PFR groups any evidenceof endophthalmitis or anyother ocular infection in either of the eyes or current treatment for serious systemic infection at the time of examination were excluded. Vitreous (300 μl) was collected from the PFR individuals (n = 6) including 3 individuals with non-PFR uveitis who were scheduled for pars plana vitrectomy/vitreous biopsy which is the normal procedure followed during the treatment of PFR (S1 Table). The treatment of the patients was symptomatic and was managed by administering oral antivirals, antibiotics, and oral steroids. All the patients were subjected to a vitreous biopsy which was performed in the operating rooms under full aseptic conditions by trained vitreoretinal surgeons. There was no difference in preparation for PPV and Vitreous Biopsy. The vitreous samples collected from the two study groups was stored at -80°C until used. This study was approved by the Institutional review board (LV Prasad Eye Institute, Hyderabad) for 2 years (from 1^st^ September 2017 to 31^st^ August 2019) with the ethics reference number LEC 09-17-079. All the samples were collected during the study period and written consent was obtained from the study participants. The study was designed and conducted according to the tenets of the Declaration of Helsinki.

### DNA extraction and metagenome sequencing

DNA was extracted from about 200 μl of vitreous sample by using PureLink DNA extraction kit (ThermoFisher Scientific, Mumbai India) according to the manufacturer’s protocol. The extracted DNA was quantified using Qubit fluorometer (Thermo Fisher Scientific, Carlsbad, CA, USA). The extracted nucleic acids were amplified with random hexamers using amplification kit (SeqPlex, Sigma Aldrich Chemicals Private Limited, Bengaluru, India). For Library preparation and sequencing, NEBNext Ultra DNA Library Prep Kit for Illumina Nextseq 500 PE sequencing protocol was followed using paired-end sequencing with 2x150bp chemistry on the Illumina Nextseq 500 platform. Care was taken to avoid microbial contamination from the environment by carrying out all the steps such as sample preparation, DNA extraction, PCR and whole genome amplification procedures in a dedicated BSL2 laminar flow hood. The laboratory where the experiments were conducted was UV sterilised prior to use. The equipment used was surface sterilised and the water used as control was autoclaved twice. Sterile water was used as a negative control instead of template DNA in PCR and consistently amplification was negative implying lack of contaminating DNA. No sequences could be generated from the negative controls.

### Fungal metagenome analysis

FASTQ files of the raw reads were generated for all the 24 samples that were sequenced. These raw sequence reads were analysed for quality parameters such as read length, phred quality score (<25), GC content and presence of ambiguous bases. The sequencing adapters from the raw sequences were trimmed by using trim-galore (version 0.4.0; Felix Krueger) and Cutadapt version 1.2 [21]. Subsequently, all reads were subjected to FastQC (version 0.11.3) tool which helped in identifying reads with quality score greater than Q25. Human genome sequences were removed by using decontam (https://github.com/benjjneb/decontam) and Bowtie 2 tools using NCBI-GRCh38 as reference genome. Reads were assigned to fungal taxa using a taxonomic classifier ‘What’s in my Pot’ (WIMP) [22]. For this the trimmed, filtered, FASTQ reads were subjected to the workflow of EPI2ME in the Oxford Nanopore Technologies [23]. Post taxonomy assignment, Meta Genome Analyser (MEGAN) v 5.11.3 was used for comparative analysis of the samples and generation of the fungal microbiome biome file for the 24 samples. The biome data file comprising abundance of all samples at genera level is provided in S2 Table. The metagenomic sequencing reads were deposited with National Center for Biotechnology Information (NCBI) and are accessible in a BioProject with accession number PRJNA646315.

### Statistical analysis

The vegan package in R (http://vegan.r-forge.r-project.org/) was used to generate rarefaction curves and for quantifying diversity indices. Genera with a mean abundance >0.002% were used for the analysis. Alpha diversity indices viz., Shannon diversity, Simpson index and Observed number of genera were calculated and the degree of variation in the fungal diversity of VC and PFR group was ascertained. Unpaired t test was conducted by using GraphPad Prism to determine statistical significance of the alpha diversity indices (https://www.graphpad.com/quickcalcs/ttest2/). Significantly different taxa in both VC and PFR groups at phylum and genus level were identified by a non-parametric, Wilcoxon signed rank test (with P<0.05 as significant). For this analysis only 6 patient samples of PFR group (PFR01 to PFR06) (S1 Table) that were clinically identified as post fever retinitis were included. These samples also tested PCR negative for the viral etiology (HSV, CMV and VZV). For visualising the relative abundances based clustering of the genera, a rank-sum normalised heatmap was generated for the fungal genera of both the cohorts. Principal co-ordinate analysis (PCoA) plot was generated for the 24 fungal microbiomes using ade4 package in R (v3.2.5) by employing Jensen–Shannon divergence distance metric. K-means clustering (k = 2) was employed to identify VC and PFR clusters on the PCoA plot.

### Correlation Network analysis of fungal genera

CoNet [24] is a Cytoscape [25] plugin that was used to detect interactive networks of the fungal microbiome in VC and PFR+ groups independently. Spearman correlation coefficient (r) was used to analyse the interactions among the different fungi (mutual exclusions/negative and co-presence/positive interactions) at the genus level.

## Results

### Total reads and taxonomic assignment of mycobiomes

**Fig 1:**
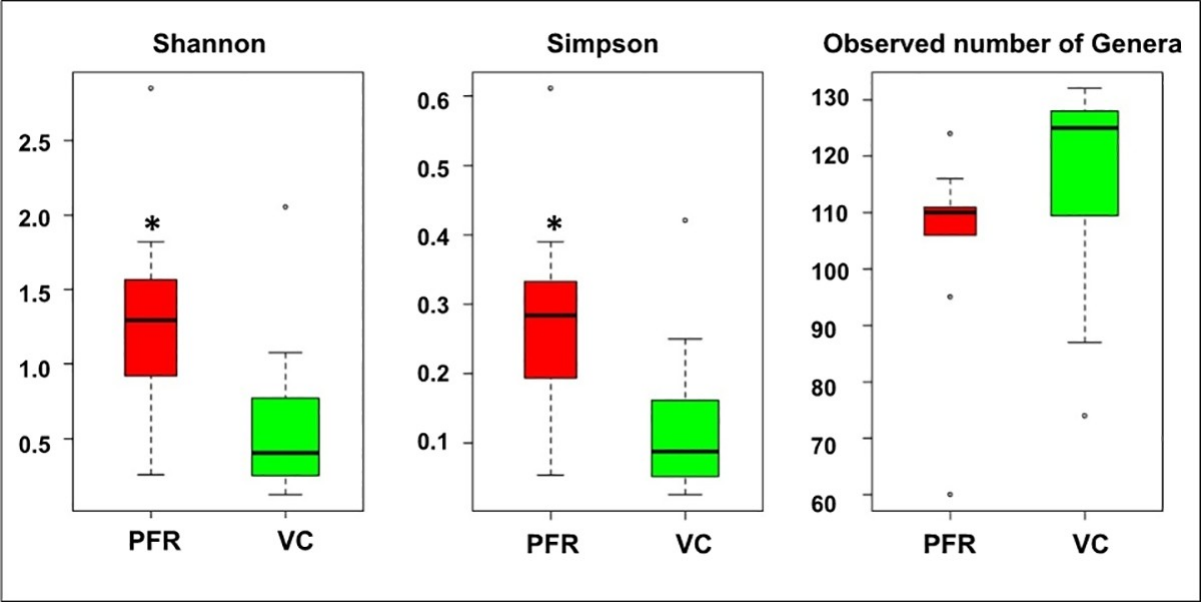
Alpha diversity indices of mycobiomes in the vitreous fluid of control (VC, n = 15) and post fever retinitis + non-PFR uveitis (PFR+, n = 9) groups. Median values (horizontal line) and interquartile ranges of the diversity indices are depicted in the box plots.

A total of 105.5 million reads were generated for all the 24 vitreous samples of control (VC, n = 15) and post fever retinitis + non-PFR uveitis (PFR+, n = 9) groups. Reads assigned to fungi comprised a total of 6.3127 million reads with 359506 and 102233 reads assigned to fungi in the control and PFR+ groups respectively (Table 1). Rarefaction curves plotted for all the 24 mycobiomes showed a tendency towards saturation indicating that majority of the fungal diversity was identified at the genera level (S1 Fig). Alphadiversity analysis showed significant difference in Simpson and Shannon diversity indices (Fig 1). While the Observed number of Genera were not statistically different between VC and PFR+ groups.

**Table 1:** Number of fungal reads generated from vitreous fluid of control (VC, n = 15) and post fever retinitis+ non-PFR uveitis (PFR+, n = 9) groups.

Samples	Total/ Average	Reads in millions (Q>25)	Reads assigned to Fungi
VC	Total	58.1	5392599
	Average	3.87	359506
PFR	Total	47.4	920101
	Average	5.27	102233
VC+PFR	Total	105.5	6312700
	Average	4.39	263029

### Relative abundance at the phyla and genera level

**Fig 2:**
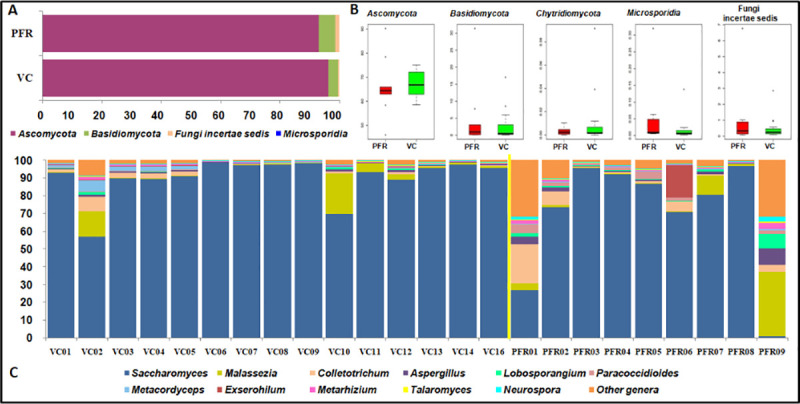
Relative abundance of different fungal phyla (A and B) and fungal genera (C) in the vitreous fluid of control (VC, n = 15) and post fever retinitis + non-PFR uveitis (PFR+, n = 9) groups. The yellow line in ‘C’ separates VC and PFR groups. Median values (horizontal line) and interquartile ranges have been depicted in the boxplots of ‘B’.

The relative abundance of the fungal taxa in control and post fever retinitis groups are summarised at both the phyla and genera level. At phyla level, *Ascomycota*, *Basidiomycota*, *Chytridiomycota* and *Microsporidia* were detected in both control (VC) and in post fever retinitis + non-PFR uveitis (PFR+) groups (Table 2). Reads assigned to phylum *Ascomycota* were predominantly present in both control and post fever retinitis groups with a mean abundance of 96.2% (range 76.9 to 99.3%) and 93.06% (range 60.25 to 99.6%) in the VC and PFR cohorts. These changes at phyla level in both VC and PFR group were not significantly different (Fig 2A and 2B). Further, the ratio of *Basidiomycota* to *Ascomycota* in VC and PFR+ is 3.4 and 6.0 respectively. At the genera level, the number of genera detected in VC and PFR+ were similar with 134 and 133 genera respectively. In addition it was observed that the most abundant top 10 genera *Saccharomyces*, *Malassezia*, *Colletotrichum*, *Aspergillus*, *Lobosporangium*, *Paracoccidiodes*, *Exserohilum*, *Metacordyceps* and *Talaromyces* were shared between all the 24 vitreous samples analysed (S3 Table; Fig 2C). Fungi incertae sedis that comprise fungi that could not be classified, undefined or unknown accounted for a mean abundance of 0.47% in the control and 1.27% in the PFR+ groups.

**Table 2:** Relative abundance of fungal phyla in the vitreous of control (VC, n = 15) and post fever retinitis + non-PFR uveitis (PFR+, n = 9) groups.

Sl. No	Phyla	Phyla Abundance in VC		Phyla Abundance in PFR+	
		Mean	Range	Mean	Range
1	*Ascomycota*	96.2162	76.9–99.3	93.05778	60.25–99.6
2	*Basidiomycota*	3.286153	0.14–21.84	5.604287	0.22–32.42
3	*Chytridiomycota*	0.013521	0–0.015	0.005574	0–0.02
4	*Microsporidia*	0.012305	0.0022–0.033	0.061571	0.008–0.33
5	Fungi incertae sedis	0.471823	0.19–1.42	1.270791	0.12–6.98

### Differentially abundant fungal genera

**Fig 3:**
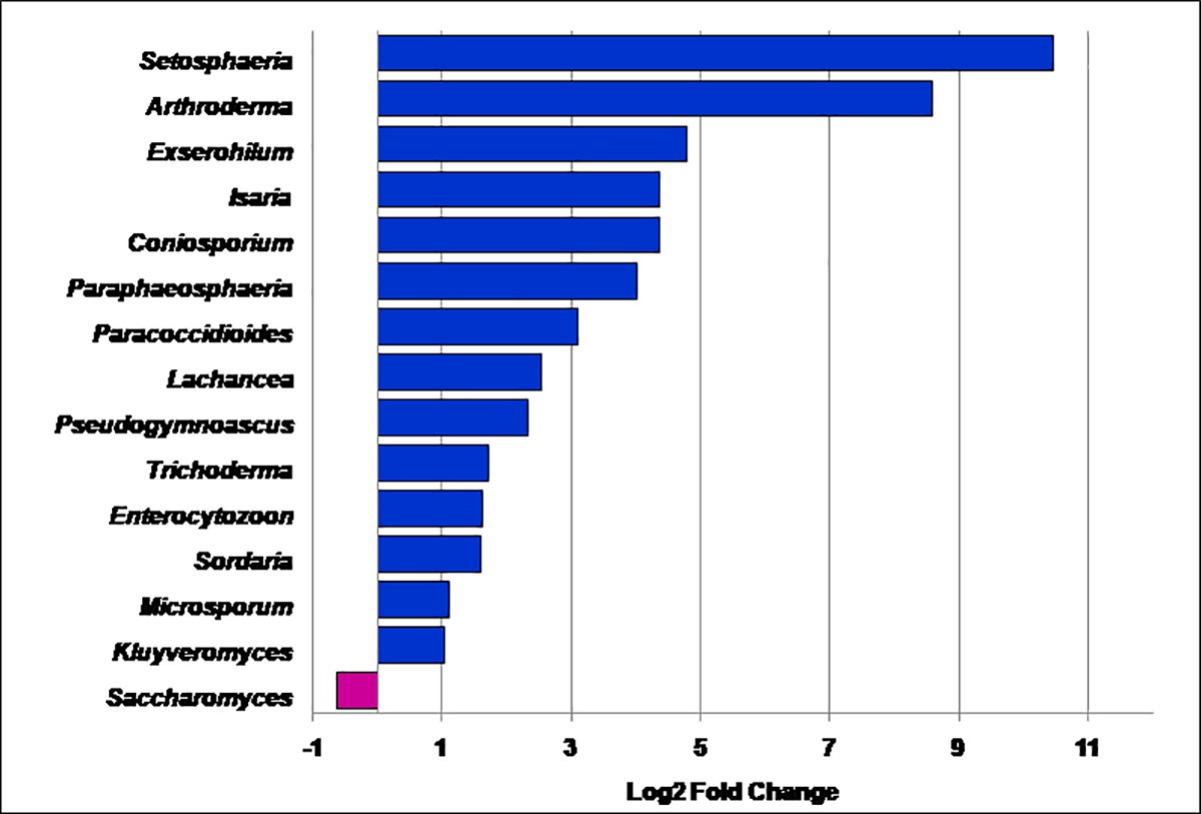
Discriminating fungal genera exhibiting significant (P<0.05) differential abundance in the vitreous fluid of control (VC, n = 15) and post fever retinitis (PFR, n = 6) groups. The horizontal bars represent the statistically significant genera as Log2 fold change. Purple and blue colour bars indicate increase in the relative abundance of the fungal genera in VC group and PFR group respectively. Genera *Nectria* and *Nematocida* were present only in VC group.

Wilcoxon signed rank test was performed between PFR group (n = 6) and control (VC) group (n = 15) to measure the significant differences in abundance at genera level (P<0.05). This resulted in identifying seventeen fungal genera that were significantly differentially abundant in post fever retinitis group compared to control group (Table 3). Out of the 17 differentially abundant genera, 14 genera were increased in abundance in PFR group and 3 genera were increased in VC group (Fig 3). Significant differences in the relative abundance of the 17 fungal genera in VC and PFR groups was also clearly visualised using boxplots (S2 Fig). Discriminative genera like *Paracoccidioides*, *Saccharomyces*, *Trichoderma* and *Kluyveromyces* were present in all the samples of VC and PFR groups. Further genera *Nectria* and *Nematocida* were present only in VC group. At the same time when all the 9 samples of PFR group were included for the analysis, 18 discriminative genera were identified (S4 Table). 13 out of 18 discriminative genera viz., *Setosphaeria*, *Arthroderma*, *Isaria*, *Paracoccidiodes*, *Sordaria*, *Nectria*, *Saccharomyces*, *Exserohilum*, *Nematocida*, *Microsporum*, *Trichoderma*, *Pseudogymnoascus and Kluveromyces* were common to the discriminative genera identified using only the 6 post fever retinitis patient samples.

**Table 3:** Discriminative fungal genera in the vitreous fluid of control (VC) and post fever retinitis (PFR) groups (P<0.05).

Genera	Mean abundance in VC	Present in number of samples (n = 15)	*Mean abundance in PFR	Present in number of samples (n = 9)	*p_value
*Setosphaeria*	0.000522	1	1.136	8	0.0001
*Arthroderma*	0.001621	1	0.953	8	0.0001
*Isaria*	0.000352	1	0.011	8	0.001
*Paracoccidioides*	0.153317	15	1.818	9	0.001
*Pseudogymnoascus*	0.007939	14	0.058	9	0.023
*Saccharomyces*	61.58861	15	56.8	9	0.003
*Sordaria*	0.011009	14	0.049	9	0.002
*Trichoderma*	0.054328	15	0.253	9	0.023
*Coniosporium*	0.0022	12	0.448	6	0.035
*Enterocytozoon*	0.01	15	0.033	9	0.023
*Exserohilum*	0.063153	14	2.35	1	0.033
*Kluyveromyces*	0.013827	15	0.041	9	0.036
*Lachancea*	0.0075	14	0.043	8	0.008
*Microsporum*	0.057128	15	0.17	4	0.023
*Nectria*	0.005494	12	0	0	0.004
*Nematocida*	0.000226	9	0	0	0.036
*Paraphaeosphaeria*	0.03	14	0.487	9	0.018

*Analysis includes only the 6 post fever retinitis samples.

**Fig 4:**
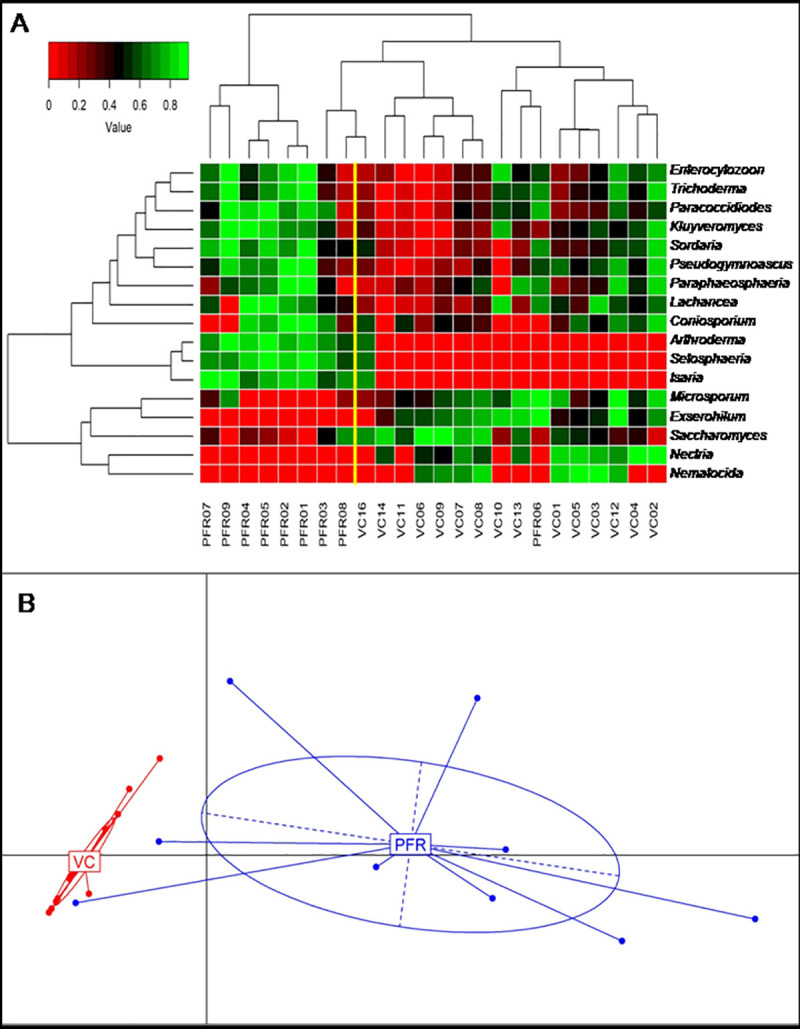
Heat map of discriminative fungal genera in the mycobiomes of vitreous fluid of control (VC, n = 15) and post fever retinitis + non-PFR uveitis (PFR+, n = 9) groups (P<0.05). A. Rank normalised abundances of the genera were scaled from 0 to 1 that are depicted by red and green colour respectively in the heat map. B. Principal co-ordinate analysis of fungal genera between control group (VC) and post fever retinitis + non-PFR uveitis (PFR+) group.

Rank normalised hierarchal clustering based heatmap analysis was done for all the 24 samples using the 17 discriminative genera (Table 3; Fig 4A). Heatmap showed two distinct clusters differentiating the control (VC) and post fever retinitis + non-PFR uveitis (PFR+) groups. However one sample from control group (VC16), clustered with PFR+ group while a sample from PFR+ group (PFR06) clustered with control group (Fig 4A). Using Jensen-Shannon Divergence (JSD) approach principal coordinate analysis was performed for all the 24 samples of control (VC) and PFR+ groups. This showed two distinct clusters for both control (VC) and post fever retinitis + non-PFR uveitis (PFR) groups (Fig 4B). All the nine samples from post fever retinitis group + non-PFR uveitis formed a separate cluster from the 15 control samples suggesting the divergence in their mycobiomes. It is worthwhile to mention that the PFR+ group also included 3 individuals viz., PFR07, PFR08 and PFR09 who were identified as having non-PFR uveitis but nevertheless in the Principal co-ordinate analysis they formed a single group implying that the mycobiomes of PFR and non-PFR uveitis are not different.

### Network analysis

**Fig 5:**
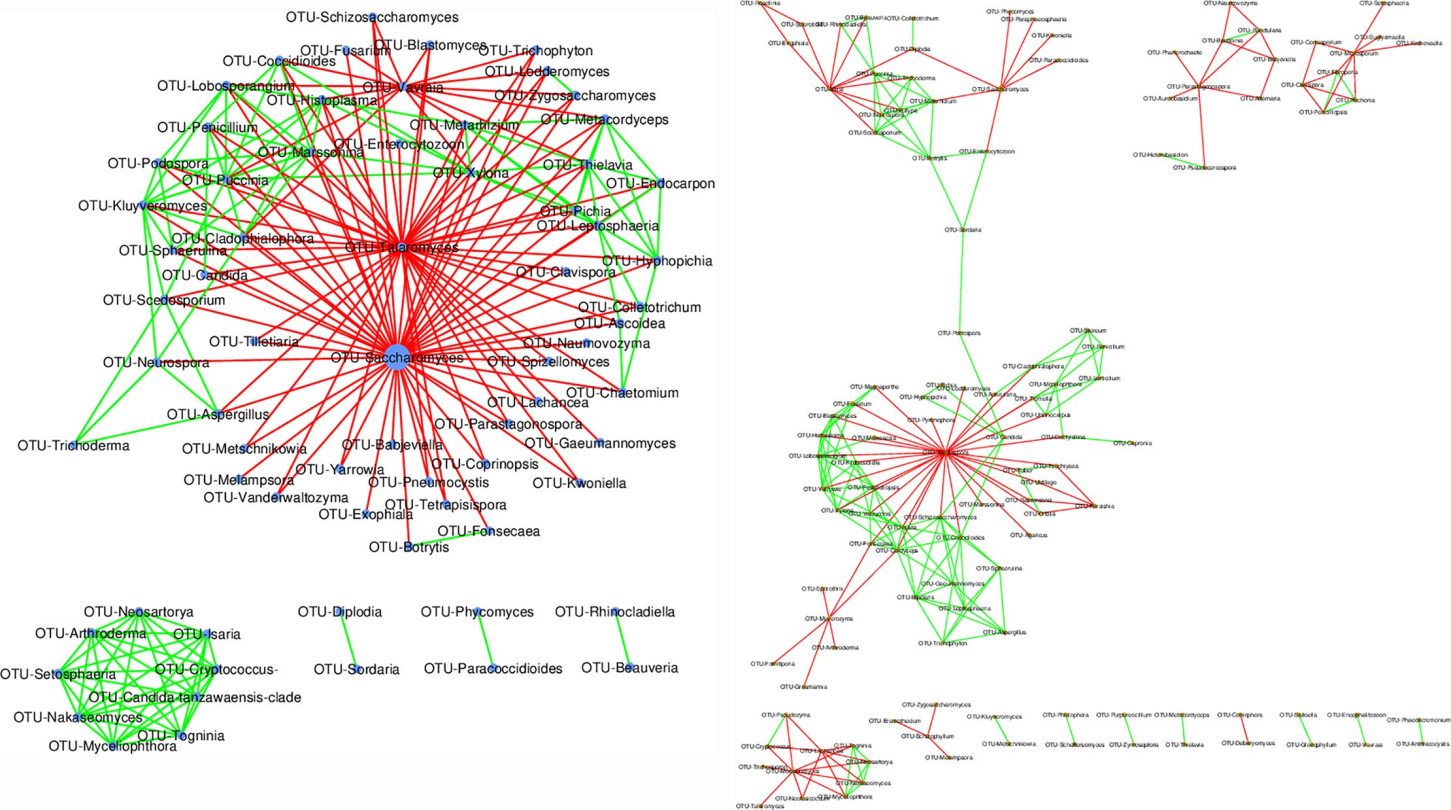
CoNet network analysis of fungal genera that co-occur in the mycobiomes of vitreous fluid of control (VC, n = 15) (A) and post fever retinitis + non-PFR uveitis (PFR+, n = 9) groups (B) (P<0.05). Nodes indicate the fungal genera. Green colour edges indicate positive interaction and red colour edges indicate negative interaction.

Fungal-fungal interactions within the control group and PFR+ group were inferred through Cytoscape generated CoNet interactions. A total of 69 of the 134 genera showed fungal-fungal interactions in the control group. Eleven genera viz., *Saccharomyces*, *Talaromyces*, *Valvraia*, *Marssonina*, *Puccinia*, *Kluyveromyces*, *Xylona*, *Histoplasma*, *Leptosphaeria*, *Metarhizium* and *Thielavia* (S4 Table) showed >10 interactions. Two major network hubs were formed by *Saccharomyces* and *Talaromyces* which showed 49 and 43 negative interactions respectively (S5A Table; Fig 5A). Positive interactions were absent for these genera. On the contrary from PFR+ group, 124 genera out of 133 genera showed more than 1 interaction with other genera (S5B Table and Fig 5B). However, only 4 genera viz., *Torulaspora*, *Cordyceps*, *Isaria* and *Mixia* showed more than 10 interactions with other genera. Genus *Torulaspora* forms the largest cluster in PFR+ group which had 31 negative interactions with other fungal genera.

## Discussion

Alterations in the bacterial microbiome have been implicated in human health and disease. However, similar studies implicating fungal microbiomes (mycobiomes) in health and disease are limited. Taking cue from theses bacterial microbiome studies there has been an increased interest in studying mycobiomes in different niches of the human body such as skin [26], oral cavity [27], gut [28–31] and ocular surface [9] in healthy individuals and in individuals with diseases like urinary tract diseases [32], Inflammatory bowel disease [33], respiratory diseases [34], ocular diseases like keratitis and uveitis [29, 35] etc. These studies showed dysbiotic changes in the mycobiomes under diseased conditions and also revealed that certain fungal species exist at low levels without causing disease but may become pathogenic under immune-compromised conditions [36]. Compared to the above human niches that have been sampled for fungi, studies on the eye are rare [37, 38] and even more rare are studies analysing the mycobiome of the vitreous fluid of the eye [1, 11, 39]. Further, detection of fungi in intraocular fluids secondary to systemic infection is extremely rare and the causative agents are often difficult to cultivate. Therefore, in the present study whole metagenomic sequencing approach was employed to demonstrate the mycobiome in 24 vitreous samples that included samples from healthy controls (VC, n = 15) and individuals with post fever retinitis + non-PFR uveitis (PFR+, n = 9).

Our results indicated that fungi affiliated to the phyla *Ascomycota* and *Basidiomycota* were the predominant phyla in the vitreous fluid of the healthy controls and PFR+ group. (Table 2 and Fig 2). That these two phyla were also reported as being predominant on the ocular surface [9, 10] and in the vitreous fluid of endophthalmitis patients [11]. We also observed that the ratio of the phyla *Basidiomycota* to *Ascomycota* increased in PFR+ group compared to VC group. This observation may be relevant considering that earlier studies had indicated that in Inflammatory bowel disease, increase in the ratio of *Basidiomycota* to *Ascomycota* is critical for the disease pathogenesis [33]. At the genera level, the top 10 genera viz., *Saccharomyces*, *Malassezia*, *Colletotrichum*, *Aspergillus*, *Paracoccidiodes*, *Exserohilum*, *Lobosporangium*, *Metacordyceps* and *Talaromyces* were assigned to 85% of the total fungal reads. It is difficult to ascertain whether this abundance status is in way related to the health status of the individual. But, some interpretation could be made based on changes in the abundance with the diseased state. The mean abundance of the genus *Saccharomyces*, a gut commensal [40] was significantly higher in control group compared to PFR group. This may be of an advantage to the healthy control group since the genus *Saccharomyces* is known to positively influence the immune system to better cope with secondary infection [41]. At the same time it was also observed that the abundance of the potential human pathogens viz., *Colletotrichum* [42], *Aspergillus* [1], *Paracoccidiodes* [43], *Exserohilum* [44], were high in PFR group compared to control (S3 Table). The analysis also indicated that VC and PFR groups could be discriminated based on 17 different genera and interestingly the functional attributes of these discriminative genera vis a vis the host could be deduced from literature (Table 4). Pathogens such as *Setosphaeria*, *Arthroderma*, *Paracoccidiodes*, *Exserohilum*, *Microsporum*, *Trichoderma*, *Pseudogymnoascus and Kluyveromyces* were all increased in PFR group compared to control group (Table 4; Fig 3). In addition a few non- pathogenic or commensal fungi such as *Isaria*, *Sordaria*, *Komagataella* and *Fomitiporia* were also increased in PFR group (Table 4; Fig 3). Among the above pathogens *Setopsphaeria*, *Trichoderma* and *Kluyveromyces* were increased in patients having fungal keratitis [10] (S6 Table). Earlier studies had also identified *Setosphaeria*, *Exserohilum* and *Trichoderma* in the vitreous fluid in endophthalmitis patients [11]. Besides the above pathogens, *Fusarium* and *Candida* the common ocular pathogens were also identified in the vitreous fluid of VC and PFR cohorts but their abundance in the two groups were not significantly different. This study demonstrates differences in the relative abundance of several fungi in the vitreous fluid of VC and PFR group. Based on these discriminative fungi VC and PFR mycobiomes could be separated into two distinct clusters both by heatmap and principal co-ordinate analysis implying that the vitreous fluid mycobiomes in PFR group is distinct from the control (VC) group. Although, the individuals in the PFR group had no clinical history of fungal infection, increase in the mean abundance of fungal genera in PFR group is difficult to explain.

**Table 4:** Attributes of the discriminative fungal genera in the vitreous of Control (VC, n = 15) and post fever retinitis (PFR, n = 6) mycobiomes.

Genera	Attribute	Increase/Decrease in PFR	Reference
*Setosphaeria*	Pathogenic	Increased in PFR	[45]
*Arthroderma*	Pathogenic	Increased in PFR	[46]
*Isaria*	Non pathogenic	Increased in PFR	_
*Paracoccidioides*	Pathogenic	Increased in PFR	[47]
*Sordaria*	Biocontrol/antifungal	Increased in PFR	[48]
*Nectria*	Opportunistic pathogen	Decreased in PFR	[49]
*Saccharomyces*	Probiotic yeast in gut	Decreased in PFR	[50]
*Exserohilum*	Pathogenic	Increased in PFR	[44]
*Enterocytozoon*	Pathogenic	Increased in PFR	[51]
*Nematocida*	_	Decreased in PFR	_
*Coniosporium*	Pathogenic	Increased in PFR	[52]
*Microsporum*	Pathogenic	Increased in PFR	[53]
*Lachancea*	-	Increasedin PFR	_
*Paraphaeosphaeria*	_	Increased in PFR	_
*Trichoderma*	Opportunistic pathogen	Increased in PFR	[54]
*Pseudogymnoascus*	Pathogenic	Increased in PFR	[55]
*Kluyveromyces*	Opportunistic pathogen	Increased in PFR	[56]

CoNet network analysis of the vitreous fluid mycobiomes also indicated that the mycobiomes based on their interaction pattern are very distinct in the VC and post fever retinitis + non-PFR uveitis (PFR+) groups. For instance, in the VC group 42.2% of the total fungal genera showed fungal-fungal interactions compared to the 89.2% of the genera in PFR+ group implying that the PFR+ group is more interactive. Further, genera that exhibited interactions with more than 10 fungal genera were identified as hub genera and in VC and PFR+ groups the number of hubs identified include 11 and 4 respectively. Out of the 11 hub genera in VC group, two hubs were formed by discriminative genera such as *Kluyveromyces* and *Saccharomyces*. *Kluyveromyces* is an opportunistic pathogen [56], that showed 10 positive interactions and 2 negative interactions with other fungal genera (S5A Table). Further, *Kluyveromyces* exhibited 4 positive interactions with human pathogenic fungi and a negative interaction with genus *Saccharomyces*. Besides, *Saccharomyces* is a commensal organism [40] that formed the major hub in control group and interacted negatively with 49 other fungal genera out of which 24 are known human pathogens. Thus it would mean that in the healthy control group *Saccharomyces* may confer beneficial effects. In contrast in PFR+ group *Saccharomyces* negatively interacted only with 4 human pathogenic fungi, thus implying that the beneficial effects that were conferred in VC group may not exist in PFR+ group. In addition genus *Isaria* is the only discriminative genera that formed a hub in PFR+ group. Genus *Isaria* is a non pathogenic fungi that exhibited 5 positive and one negative interaction with human pathogenic fungi. On the whole it appears that the networks generated for PFR+ group lack the prominent hubs that could negate the pathogenic fungal interactions as observed in VC group.

Increasing evidences suggest the importance of microbiome studies as a potential modifiable factor in therapeutics [57, 58]. The results of the present study may not be directly impacting the clinical outcome. However the observations of the study reveal that there was an increase in the abundance of pathogenic fungi in patient samples compared to the control. Conversely, the abundance of few fungal genera that may have beneficiary effect were decreased in the patient cohort. Another important observation that could be made from the network analysis is that microbial interactions are crucial in maintaining the homeostasis and this was disrupted under the diseased condition. All these evidences put together imply that the changes in the mycobiome composition in retinitis individuals were significant but it would be difficult to assess its impact on the treatment outcomes from the available data.

The study involving generation of mycobiomes from the vitreous samples has a few limitations and biases. One of the limitations is the lack of vitreous sample from healthy individuals, as controls, for want of ethical clearance. The study has less number of individuals recruited in PFR group due to rareness of the conditions and due to ethical compliance for vitreous biopsy. Further, the collection of vitreous is an invasive process involving the use of a syringe to pierce the ocular surface, access the intraocular space and retrieve the vitreous. This procedure though accomplished in an operation theatre does not ensure possible contamination by the ocular surface mycobiome. Therefore analysis of the ocular surface mycobiome from all the participants may be required for comparison with vitreous. Since in the present study the ocular surface samples were not collected, the data of the present study was compared with the ocular surface mycobiome from published literature [9–11]. The mycobiome from blood in control and PFR group participants could have enhanced the understanding of the mycobiome penetration into the eye. Simultaneously mycobiome from the individuals with fungal retinitis would have helped in comparing the data to understand the changes in the vitreous due to fungal pathogens.

## Conclusions

The findings of the study point towards an imbalance in the vitreous mycobiome in PFR group compared to control group. Alpha diversity indices (Simpson and Shannon diversity indices) between VC and PFR+ group were significantly different. VC and PFR+ cohorts could also be discriminated based on the ratio of *Basidiomycota* to *Ascomycota* and the abundance of several genera. Significant decrease in the relative abundance of predominant commensal genus *Saccharomyces* and increase in the abundance of 9 fungal human pathogens viz., *Setosphaeria*, *Arthroderma*, *Enterocytozoon*, *Exserohilum*, *Paracoccidiodes*, *Pseudogymnoascus*, *Trichoderma*, *Kluveromyces* and *Microsporum* signifies the possible role of these genera in the pathogenesis of PFR. Thus the study provides evidence that the mycobiome of the vitreous fluid in post fever retinitis individuals is distinct compared to the healthy controls.

## Supporting information

S1 Fig Rarefaction curves of mycobiomes from vitreous fluid of controls ((VC, n = 15) and post fever retinitis + non-PFR uveitis (PFR+, n = 9) groups. Curves were plotted for the genera having a mean abundance > 0.002%.
(TIF)

S2 Fig Boxplot histograms of 17 discriminative fungal genera in the mycobiomes of vitreous fluid of control (VC, n = 15) and post fever retinitis + non-PFR uveitis (PFR+, n = 9) groups (P<0.05). Median values (horizontal line) and interquartile ranges have been depicted in the plots.
(TIF)

S1 Table Sample collection details of control samples (VC, n = 15) and post fever retinitis+ non-PFR uveitis samples (PFR+, n = 9). (DOCX)

S2 Table Mycobiome biome file of control samples (VC, n = 15) and post fever retinitis samples + non-PFR uveitis (PFR+, n = 9). (XLSX)

S3 Table Abundance of fungal genera in the vitreous of Control (VC) and post fever retinitis + non-PFR uveitis (PFR+) samples. Genera having a mean abundance of >0.002% are listed in the table.
(DOCX)

S4 Table Discriminative fungal genera in the vitreous fluid of control (VC, n = 15) and post fever retinitis (PFR, n = 6) groups (P<0.05). (DOCX)

S5 Table A. Co-occurrence network analysis of control (VC) group determining the number of positive and negative interactions among the fungal genera. B. Co-occurrence network analysis of PFR+ group determining the number of positive and negative interactions among the fungal genera.
(DOCX)

S6 Table Presence or absence of discriminative genera of the present study in other ocular studies. (DOCX)
