# Supplementary figures and images for: Mycobiome changes in the vitreous of post fever retinitis patients
Source: PLoS One. 2020 Nov 19;15(11):e0242138. doi: 10.1371/journal.pone.0242138 (PMC7676714; doi:10.1371/journal.pone.0242138)

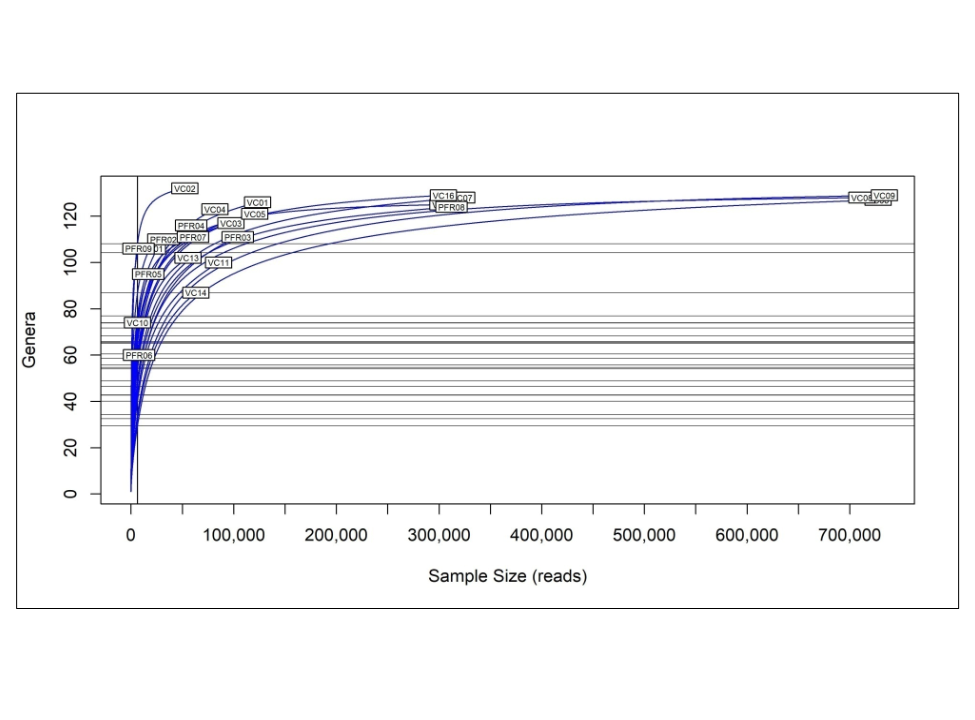

Supplement: S1 Fig — Curves were plotted for the genera having a mean abundance > 0.002%. (TIF) [file pone.0242138.s001.tif]

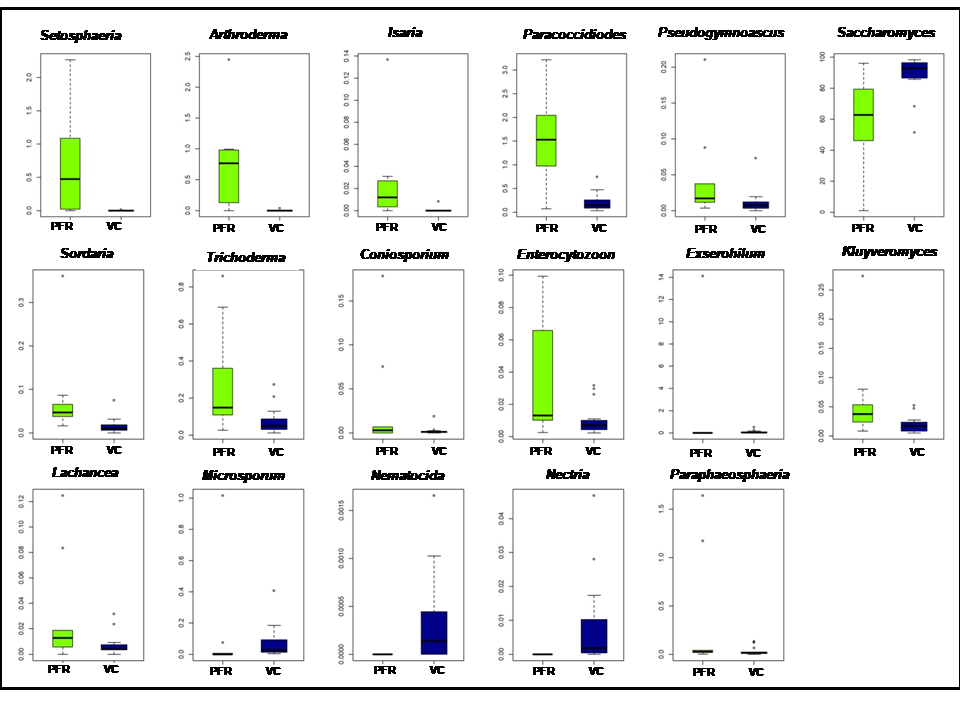

Supplement: S2 Fig — Median values (horizontal line) and interquartile ranges have been depicted in the plots. (TIF) [file pone.0242138.s002.tif]
