# Supplementary material for: Mycobiome changes in the vitreous of post fever retinitis patients
Source: PLoS One. 2020 Nov 19;15(11):e0242138. doi: 10.1371/journal.pone.0242138 (PMC7676714; doi:10.1371/journal.pone.0242138)
Supplement: S1 Table — (DOCX) [file pone.0242138.s003.docx]

S1 Table. Sample collection detaills of control samples (VC, n=15) and post fever retinitis + non-PFR uveitis samples (PFR+, n=9).

| **Sl. No.** | **Sample ID** | **Age  (years)** | **Gender** | **State** | **Vitreous sample collection procedure** | **History of Retinitis** |
| --- | --- | --- | --- | --- | --- | --- |
| 1 | VC01 | 61 | Female | Telangana | Pars plana vitrectomy | nil |
| 2 | VC02 | 46 | Female | Karnataka | Pars plana vitrectomy | nil |
| 3 | VC03 | 63 | Male | Madhya Pradesh | Pars plana vitrectomy | nil |
| 4 | VC04 | 47 | Male | West Bengal | Pars plana vitrectomy | nil |
| 5 | VC05 | 36 | Male | Andhra Pradesh | Pars plana vitrectomy | nil |
| 6 | VC06 | 36 | Male | Andhra Pradesh | Pars plana vitrectomy | nil |
| 7 | VC07 | 22 | Male | West Bengal | Pars plana vitrectomy | nil |
| 8 | VC08 | 57 | Male | Telangana | Pars plana vitrectomy | nil |
| 9 | VC09 | 67 | Female | Andhra Pradesh | Pars plana vitrectomy | nil |
| 10 | VC10 | 69 | Male | Maharashtra | Pars plana vitrectomy | nil |
| 11 | VC11 | 54 | Male | Telangana | Pars plana vitrectomy | nil |
| 12 | VC12 | 56 | Male | Telangana | Pars plana vitrectomy | nil |
| 13 | VC13 | 37 | Male | Telangana | Pars plana vitrectomy | nil |
| 14 | VC14 | 33 | Female | Andhra Pradesh | Pars plana vitrectomy | nil |
| 15 | VC16 | 42 | Male | Telangana | Pars plana vitrectomy | nil |
| 16 | PFR01 | 35 | Female | Maharashtra | Pars plana vitrectomy | Post febrile illness |
| 17 | PFR02 | 32 | Male | Telangana | Vitreous biopsy | Post Typhoid fever |
| 18 | PFR03 | 20 | Female | Karnataka | Vitreous biopsy | Post febrile illness |
| 19 | PFR04 | 42 | Male | Telangana | Vitreous biopsy | Post febrile illness |
| 20 | PFR05 | 39 | Male | Telangana | Vitreous biopsy | Post Typhoid fever |
| 21 | PFR06 | 40 | Female | Andhra Pradesh | Vitreous biopsy | Post febrile illness |
| 22 | PFR07 | 46 | Male | Telangana | Pars plana vitrectomy | Taxoplasma retinitis |
| 23 | PFR08 | 26 | Male | Telangana | Pars plana vitrectomy | Tuberculosis retinitis |
| 24 | PFR09 | 46 | Male | Telangana | Vitreous biopsy | Viral retinitis |
