# Supplementary material for: Mycobiome changes in the vitreous of post fever retinitis patients
Source: PLoS One. 2020 Nov 19;15(11):e0242138. doi: 10.1371/journal.pone.0242138 (PMC7676714; doi:10.1371/journal.pone.0242138)
Supplement: S3 Table — Genera having a mean abundance of >0.002% are listed in the table. (DOCX) [file pone.0242138.s005.docx]

S3 Table. Abundance of fungal genera in the vitreous of Control (VC) and post fever retinitis + non-PFR uveitis (PFR+) samples. Genera having a mean abundance of >0.002% are listed in the table

| Sl. No. | Genera | VC_mean | PFR_mean |
| --- | --- | --- | --- |
|  | *Saccharomyces* | 88.30312 | 57.47115 |
|  | *Malassezia* | 3.271687 | 5.60109 |
|  | *Colletotrichum* | 1.377308 | 2.705649 |
|  | *Aspergillus* | 0.575314 | 1.800044 |
|  | *Paracoccidioides* | 0.214291 | 1.497553 |
|  | *Exserohilum* | 0.084761 | 1.567501 |
|  | *Lobosporangium* | 0.407207 | 1.237625 |
|  | *Metacordyceps* | 1.00269 | 0.133202 |
|  | *Metarhizium* | 0.300587 | 0.655208 |
|  | *Talaromyces* | 0.445466 | 0.424832 |
|  | *Setosphaeria* | 0.000807 | 0.781516 |
|  | *Arthroderma* | 0.002509 | 0.763593 |
|  | *Neurospora* | 0.135425 | 0.504168 |
|  | *Clavispora* | 0.016421 | 0.473121 |
|  | *Histoplasma* | 0.070547 | 0.367469 |
|  | *Thielavia* | 0.135757 | 0.260505 |
|  | *Blastomyces* | 0.091632 | 0.285588 |
|  | *Trichoderma* | 0.077021 | 0.282575 |
|  | *Paraphaeosphaeria* | 0.030254 | 0.328754 |
|  | *Trichophyton* | 0.198994 | 0.133661 |
|  | *Yarrowia* | 0.037684 | 0.279836 |
|  | *Scedosporium* | 0.060071 | 0.252218 |
|  | *Phycomyces* | 0.086946 | 0.21311 |
|  | *Coccidioides* | 0.073706 | 0.207895 |
|  | *Leptosphaeria* | 0.120329 | 0.151735 |
|  | *Puccinia* | 0.057755 | 0.191556 |
|  | *Hyphopichia* | 0.062294 | 0.148799 |
|  | *Pichia* | 0.065964 | 0.142916 |
|  | *Microsporum* | 0.07931 | 0.12204 |
|  | *Moesziomyces* | 0.0241 | 0.158569 |
|  | *Penicillium* | 0.05558 | 0.125077 |
|  | *Chaetomium* | 0.088399 | 0.084365 |
|  | *Tetrapisispora* | 0.032545 | 0.115049 |
|  | *Magnaporthe* | 0.035409 | 0.10429 |
|  | *Marssonina* | 0.037126 | 0.098641 |
|  | *Fusarium* | 0.035955 | 0.097843 |
|  | *Endocarpon* | 0.103932 | 0.026249 |
|  | *Schizosaccharomyces* | 0.03588 | 0.07942 |
|  | *Phialocephala* | 0.035028 | 0.077084 |
|  | *Sphaerulina* | 0.019563 | 0.079644 |
|  | *Sordaria* | 0.016175 | 0.079916 |
|  | *Xylona* | 0.031349 | 0.059345 |
|  | *Tuber* | 0.02692 | 0.062753 |
|  | *Fonsecaea* | 0.017235 | 0.068037 |
|  | *Kluyveromyces* | 0.019922 | 0.062614 |
|  | *Verruconis* | 0.01558 | 0.061477 |
|  | *Diplodia* | 0.01327 | 0.055718 |
|  | *Cladophialophora* | 0.022356 | 0.045322 |
|  | *Exophiala* | 0.01781 | 0.048632 |
|  | *Bipolaris* | 0.015558 | 0.050615 |
|  | *Sclerotinia* | 0.007648 | 0.051642 |
|  | *Filobasidiella* | 0.017175 | 0.041597 |
|  | *Eutypa* | 0.016022 | 0.040959 |
|  | *Pseudogymnoascus* | 0.011716 | 0.044783 |
|  | *Rhinocladiella* | 0.011887 | 0.044235 |
|  | *Myceliophthora* | 0.001571 | 0.054271 |
|  | *Lodderomyces* | 0.025558 | 0.029622 |
|  | *Orbilia* | 0.014178 | 0.040648 |
|  | *Naumovozyma* | 0.016449 | 0.037092 |
|  | *Ascoidea* | 0.014948 | 0.03813 |
|  | *Penicilliopsis* | 0.01986 | 0.032983 |
|  | *Melampsora* | 0.010809 | 0.040807 |
|  | *Verticilium* | 0.021922 | 0.029444 |
|  | *Botrytis* | 0.014875 | 0.034645 |
|  | *Aureobasidium* | 0.013726 | 0.035535 |
|  | *Podospora* | 0.015014 | 0.032895 |
|  | *Enterocytozoon* | 0.010708 | 0.034664 |
|  | *Candida* | 0.013192 | 0.031612 |
|  | *Beauveria* | 0.010775 | 0.032501 |
|  | *Kwoniella* | 0.011508 | 0.029022 |
|  | *Zygosaccharomyces* | 0.011622 | 0.027717 |
|  | *Lachancea* | 0.007537 | 0.030594 |
|  | *Kazachstania* | 0.013892 | 0.024161 |
|  | *Gaeumannomyces* | 0.008027 | 0.028376 |
|  | *Sporothrix* | 0.005499 | 0.028947 |
|  | *Coniosporium* | 0.002189 | 0.029958 |
|  | *Pestalotiopsis* | 0.01008 | 0.021731 |
|  | *unclassified Microsporidia* | 0.000305 | 0.030775 |
|  | *Cordyceps* | 0.016228 | 0.0148 |
|  | *Pseudozyma* | 0.00325 | 0.026494 |
|  | *Debaryomyces* | 0.012497 | 0.016931 |
|  | *Isaria* | 0.000545 | 0.026672 |
|  | *Metschnikowia* | 0.009328 | 0.017246 |
|  | *Moniliophthora* | 0.006972 | 0.017429 |
|  | *Wickerhamomyces* | 0.010623 | 0.012192 |
|  | *Dactylellina* | 0.005764 | 0.016891 |
|  | *Pyrenophora* | 0.007817 | 0.01326 |
|  | *Eremothecium* | 0.005 | 0.015367 |
|  | *Agaricus* | 0.005505 | 0.013969 |
|  | *Grosmannia* | 0.004172 | 0.015126 |
|  | *Pneumocystis* | 0.007058 | 0.011699 |
|  | *Fomitiporia* | 0.004361 | 0.01402 |
|  | *Zymoseptoria* | 0.003607 | 0.013981 |
|  | *Cryptococcus* | 0.000327 | 0.01711 |
|  | *Pochonia* | 0.007932 | 0.008861 |
|  | *Punctularia* | 0.003732 | 0.01238 |
|  | *Vanderwaltozyma* | 0.006722 | 0.009241 |
|  | *Torulaspora* | 0.005765 | 0.009715 |
|  | *Capronia* | 0.005042 | 0.010236 |
|  | *Neosartorya* | 0.002029 | 0.01184 |
|  | *Babjeviella* | 0.004721 | 0.008413 |
|  | *Ustilago* | 0.003166 | 0.009934 |
|  | *Wallemia* | 0.008736 | 0.004294 |
|  | *Trichosporon* | 0.000748 | 0.011379 |
|  | *Laccaria* | 0.004609 | 0.006945 |
|  | *Trametes* | 0.002892 | 0.008625 |
|  | *Phanerochaete* | 0.001918 | 0.009576 |
|  | *Rhodotorula* | 0.00302 | 0.008439 |
|  | *Spizellomyces* | 0.007701 | 0.003507 |
|  | *Komagataella* | 0.001482 | 0.00959 |
|  | *Kuraishia* | 0.000875 | 0.010025 |
|  | *Tremella* | 0.004553 | 0.006151 |
|  | *Coprinopsis* | 0.003914 | 0.006525 |
|  | *Parastagonospora* | 0.004817 | 0.005241 |
|  | *Sugiyamaella* | 0.002914 | 0.006548 |
|  | *Auricularia* | 0.00116 | 0.007815 |
|  | *Fibroporia* | 0.001499 | 0.007398 |
|  | *Anthracocystis* | 0.003836 | 0.004795 |
|  | *Nectria* | 0.008371 | 0 |
|  | *Serpula* | 0.002071 | 0.006001 |
|  | *Rasamsonia* | 0.00183 | 0.006179 |
|  | *Stereum* | 0.003316 | 0.004245 |
|  | *Cyberlindnera* | 0.001329 | 0.005687 |
|  | *Uncinocarpus* | 0.002792 | 0.004208 |
|  | *Batrachochytrium* | 0.004489 | 0.002455 |
|  | *Purpureocillium* | 0.003297 | 0.003423 |
|  | *Heterobasidion* | 0.001495 | 0.005121 |
|  | *Alternaria* | 0.004047 | 0.002373 |
|  | *Tsuchiyaea* | 0.002671 | 0.003708 |
|  | *Spathaspora* | 0.003586 | 0.002698 |
|  | *Kockovaella* | 0.001612 | 0.003918 |
|  | *Pseudocercospora* | 0.002101 | 0.003353 |
|  | *Rosellinia* | 0.00415 | 0.000956 |
|  | *Ogataea* | 0.002695 | 0.002387 |
| **Total genera** | | **134** | **133** |
