# Supplementary material for: Mycobiome changes in the vitreous of post fever retinitis patients
Source: PLoS One. 2020 Nov 19;15(11):e0242138. doi: 10.1371/journal.pone.0242138 (PMC7676714; doi:10.1371/journal.pone.0242138)
Supplement: S4 Table — (DOCX) [file pone.0242138.s006.docx]

S4 Table: Discriminative fungal genera in the vitreous fluid of control (VC, n=15) and post fever retinitis + non-PFR uveitis (PFR+, n=9) groups (P<0.05).

| Genera | Mean abundance in VC | Present in number of samples (n=15) | Mean abundance in PFR | Present in number of samples (n=9) | p_value |
| --- | --- | --- | --- | --- | --- |
| *Setosphaeria* | 0.000522 | 1 | 0.551527 | 8 | 0 |
| *Arthroderma* | 0.001621 | 1 | 0.516546 | 8 | 0 |
| *Isaria* | 0.000352 | 1 | 0.023339 | 8 | 0 |
| *Paracoccidioides* | 0.153317 | 15 | 1.050036 | 9 | 0.001 |
| *Sordaria* | 0.011009 | 14 | 0.066646 | 9 | 0.001 |
| *Nectria* | 0.005494 | 12 | 0 | 0 | 0.001 |
| *Saccharomyces* | 61.58861 | 15 | 37.44635 | 9 | 0.003 |
| *Exserohilum* | 0.063153 | 14 | 0.924101 | 1 | 0.003 |
| *Clavispora* | 0.010936 | 15 | 0.436152 | 9 | 0.003 |
| *Nematocida* | 0.000226 | 9 | 0 | 0 | 0.012 |
| *Komagataella* | 0.00098 | 10 | 0.006303 | 8 | 0.018 |
| *Microsporum* | 0.057128 | 15 | 0.075161 | 4 | 0.023 |
| *Scheffersomyces* | 0.001218 | 10 | 0.000115 | 1 | 0.027 |
| *Fomitiporia* | 0.002941 | 9 | 0.012281 | 8 | 0.03 |
| *Glarea* | 0.002183 | 13 | 0.000853 | 2 | 0.032 |
| *Trichoderma* | 0.054328 | 15 | 0.238004 | 9 | 0.035 |
| *Pseudogymnoascus* | 0.007939 | 14 | 0.038164 | 9 | 0.041 |
| *Kluyveromyces* | 0.013827 | 15 | 0.050753 | 9 | 0.048 |
