# Supplementary material for: Mycobiome changes in the vitreous of post fever retinitis patients
Source: PLoS One. 2020 Nov 19;15(11):e0242138. doi: 10.1371/journal.pone.0242138 (PMC7676714; doi:10.1371/journal.pone.0242138)
Supplement: S5 Table — A. Co-occurrence network analysis of control (VC) group determining the number of positive and negative interactions among the fungal genera. B. Co-occurrence network analysis of PFR+ group determining the number of positive and negative interactions among the fungal genera. (DOCX) [file pone.0242138.s007.docx]

S5A Table: Co-occurrence network analysis of control (VC) group determining the number of positive and negative interactions among the fungal genera

| **Genera** | **Total interactions** | **Negative interaction** | **Positive interaction** | **Sample count** |
| --- | --- | --- | --- | --- |
| *Arthroderma* | 7 | 0 | 7 | 1 |
| *Clavispora* | 2 | 2 | 0 | 15 |
| *Isaria* | 7 | 0 | 7 | 1 |
| *Kluyveromyces* | 12 | 2 | 10 | 15 |
| *Paracoccidioides* | 1 | 0 | 1 | 15 |
| *Saccharomyces* | 49 | 49 | 0 | 15 |
| *Setosphaeria* | 7 | 0 | 7 | 1 |
| *Sordaria* | 1 | 0 | 1 | 14 |
| *Trichoderma* | 3 | 0 | 3 | 15 |
| *Ascoidea* | 4 | 2 | 2 | 15 |
| *Aspergillus* | 6 | 2 | 4 | 15 |
| *Babjeviella* | 1 | 1 | 0 | 12 |
| *Beauveria* | 1 | 0 | 1 | 15 |
| *Blastomyces* | 3 | 3 | 0 | 15 |
| *Botrytis* | 2 | 1 | 1 | 15 |
| *Candida* | 3 | 2 | 1 | 15 |
| *Chaetomium* | 5 | 2 | 3 | 15 |
| *Cladophialophora* | 9 | 3 | 6 | 15 |
| *Coccidioides* | 7 | 3 | 4 | 15 |
| *Colletotrichum* | 3 | 2 | 1 | 15 |
| *Coprinopsis* | 2 | 2 | 0 | 11 |
| *Cryptococcus* | 7 | 0 | 7 | 1 |
| *Diplodia* | 1 | 0 | 1 | 14 |
| *Endocarpon* | 7 | 2 | 5 | 15 |
| *Enterocytozoon* | 1 | 1 | 0 | 15 |
| *Exophiala* | 1 | 1 | 0 | 15 |
| *Fonsecaea* | 2 | 1 | 1 | 15 |
| *Fusarium* | 2 | 2 | 0 | 15 |
| *Gaeumannomyces* | 1 | 1 | 0 | 15 |
| *Histoplasma* | 11 | 3 | 8 | 15 |
| *Hyphopichia* | 7 | 2 | 5 | 15 |
| *Kwoniella* | 2 | 2 | 0 | 13 |
| *Lachancea* | 2 | 2 | 0 | 14 |
| *Leptosphaeria* | 10 | 2 | 8 | 15 |
| *Lobosporangium* | 8 | 3 | 5 | 15 |
| *Lodderomyces* | 4 | 3 | 1 | 15 |
| *Marssonina* | 14 | 2 | 12 | 15 |
| *Melampsora* | 1 | 1 | 0 | 15 |
| *Metacordyceps* | 8 | 3 | 5 | 15 |
| *Metarhizium* | 10 | 3 | 7 | 15 |
| *Metschnikowia* | 2 | 2 | 0 | 15 |
| *Myceliophthora* | 7 | 0 | 7 | 1 |
| *Nakaseomyces* | 7 | 0 | 7 | 1 |
| *Naumovozyma* | 1 | 1 | 0 | 15 |
| *Neosartorya* | 7 | 0 | 7 | 1 |
| *Neurospora* | 6 | 1 | 5 | 15 |
| *Parastagonospora* | 2 | 2 | 0 | 14 |
| *Penicillium* | 6 | 2 | 4 | 15 |
| *Phycomyces* | 1 | 0 | 1 | 15 |
| *Pichia* | 7 | 2 | 5 | 15 |
| *Pneumocystis* | 2 | 2 | 0 | 14 |
| *Podospora* | 8 | 2 | 6 | 15 |
| *Puccinia* | 13 | 3 | 10 | 15 |
| *Rhinocladiella* | 1 | 0 | 1 | 15 |
| *Scedosporium* | 8 | 2 | 6 | 15 |
| *Schizosaccharomyces* | 3 | 3 | 0 | 15 |
| *Sphaerulina* | 5 | 2 | 3 | 15 |
| *Spizellomyces* | 2 | 2 | 0 | 12 |
| *Talaromyces* | 43 | 43 | 0 | 15 |
| *Tetrapisispora* | 2 | 2 | 0 | 15 |
| *Thielavia* | 10 | 3 | 7 | 15 |
| *Tilletiaria* | 2 | 2 | 0 | 6 |
| *Togninia* | 7 | 0 | 7 | 1 |
| *Trichophyton* | 3 | 3 | 0 | 15 |
| *Vanderwaltozyma* | 2 | 2 | 0 | 15 |
| *Vavraia* | 15 | 15 | 0 | 3 |
| *Xylona* | 11 | 3 | 8 | 15 |
| *Yarrowia* | 3 | 2 | 1 | 15 |
| *Zygosaccharomyces* | 4 | 3 | 1 | 15 |

S5B Table: Co-occurrence network analysis of PFR+ group determining the number of positive and negative interactions among the fungal genera

| **Genera** | **Total ineractions** | **Negative interaction** | **Positive interaction** | **Sample count** |
| --- | --- | --- | --- | --- |
| *Arthroderma* | 1 | 1 | 0 | 8 |
| *Clavispora* | 4 | 4 | 0 | 9 |
| *Fomitiporia* | 1 | 1 | 0 | 8 |
| *Isaria* | 11 | 1 | 10 | 8 |
| *Kluyveromyces* | 1 | 0 | 1 | 9 |
| *Microsporum* | 7 | 7 | 0 | 4 |
| *Paracoccidioides* | 1 | 1 | 0 | 9 |
| *Saccharomyces* | 7 | 7 | 0 | 9 |
| *Scheffersomyces* | 1 | 0 | 1 | 1 |
| *Setosphaeria* | 1 | 1 | 0 | 8 |
| *Trichoderma* | 5 | 1 |  | 9 |
| *Agaricus* | 1 | 1 | 0 | 7 |
| *Alternaria* | 2 | 2 | 0 | 5 |
| *Anthracocystis* | 1 | 0 | 1 | 4 |
| *Aspergillus* | 5 | 0 | 5 | 9 |
| *Aureobasidium* | 1 | 1 | 0 | 6 |
| *Auricularia* | 2 | 1 | 1 | 6 |
| *Babjeviella* | 3 | 3 | 0 | 8 |
| *Baudoinia* | 4 | 3 | 1 | 4 |
| *Beauveria* | 3 | 1 | 2 | 9 |
| *Bipolaris* | 8 | 0 | 8 | 8 |
| *Blastomyces* | 6 | 1 | 5 | 9 |
| *Botrytis* | 6 | 0 | 6 | 8 |
| *Candida* | 5 | 0 | 5 | 9 |
| *Capronia* | 1 | 0 | 1 | 6 |
| *Cladophialophora* | 4 | 1 | 3 | 7 |
| *Coccidioides* | 9 | 1 | 8 | 9 |
| *Colletotrichum* | 1 | 0 | 1 | 9 |
| *Coniophora* | 1 | 1 | 0 | 4 |
| *Coniosporium* | 2 | 2 | 0 | 6 |
| *Cordyceps* | 11 | 2 | 9 | 8 |
| *Cryptococcus* | 3 | 2 | 1 | 4 |
| *Dactylellina* | 3 | 1 | 2 | 7 |
| *Debaryomyces* | 1 | 1 | 0 | 8 |
| *Diplodia* | 3 | 2 | 1 | 9 |
| *Encephalitozoon* | 1 | 0 | 1 | 2 |
| *Enterocytozoon* | 3 | 1 | 2 | 9 |
| *Eremothecium* | 1 | 1 | 0 | 8 |
| *Eutypa* | 7 | 1 | 6 | 9 |
| *Exophiala* | 2 | 2 | 0 | 9 |
| *Fibroporia* | 4 | 2 | 2 | 5 |
| *Filobasidiella* | 7 | 1 | 6 | 8 |
| *Fonsecaea* | 5 | 2 | 3 | 8 |
| *Fusarium* | 5 | 1 | 4 | 9 |
| *Gaeumannomyces* | 8 | 0 | 8 | 8 |
| *Gloeophyllum* | 1 | 0 | 1 | 3 |
| *Grosmannia* | 1 | 1 | 0 | 7 |
| *Heterobasidion* | 1 | 0 | 1 | 5 |
| *Histoplasma* | 8 | 1 | 7 | 9 |
| *Hyphopichia* | 2 | 0 | 2 | 9 |
| *Kockovaella* | 1 | 1 | 0 | 7 |
| *Kuraishia* | 4 | 4 | 0 | 5 |
| *Kwoniella* | 1 | 1 | 0 | 9 |
| *Lachancea* | 7 | 7 | 0 | 8 |
| *Leptosphaeria* | 7 | 0 | 7 | 9 |
| *Lobosporangium* | 8 | 1 | 7 | 9 |
| *Lodderomyces* | 1 | 1 | 0 | 8 |
| *Magnaporthe* | 3 | 1 | 2 | 9 |
| *Malassezia* | 2 | 1 | 1 | 9 |
| *Marssonina* | 1 | 1 | 0 | 9 |
| *Melampsora* | 1 | 1 | 0 | 9 |
| *Metacordyceps* | 1 | 0 | 1 | 8 |
| *Metarhizium* | 7 | 1 | 6 | 9 |
| *Metschnikowia* | 1 | 0 | 1 | 9 |
| *Meyerozyma* | 6 | 6 | 0 | 5 |
| *Mixia* | 10 | 10 | 0 | 4 |
| *Moesziomyces* | 9 | 9 | 0 | 6 |
| *Moniliophthora* | 6 | 1 | 5 | 7 |
| *Myceliophthora* | 5 | 2 | 3 | 3 |
| *Nakaseomyces* | 5 | 2 | 3 | 3 |
| *Naumovozyma* | 2 | 2 | 0 | 8 |
| *Neofusicoccum* | 2 | 2 | 0 | 5 |
| *Neosartorya* | 5 | 2 | 3 | 3 |
| *Neurospora* | 7 | 1 | 6 | 9 |
| *Orbilia* | 3 | 2 | 1 | 8 |
| *Paraphaeosphaeria* | 1 | 1 | 0 | 9 |
| *Parastagonospora* | 6 | 6 | 0 | 7 |
| *Penicilliopsis* | 4 | 2 | 2 | 5 |
| *Penicillium* | 4 | 0 | 4 | 8 |
| *Pestalotiopsis* | 9 | 1 | 8 | 8 |
| *Phaeoacremonium* | 1 | 0 | 1 | 4 |
| *Phanerochaete* | 1 | 1 | 0 | 4 |
| *Phialophora* | 1 | 0 | 1 | 1 |
| *Phycomyces* | 1 | 1 | 0 | 9 |
| *Pichia* | 2 | 0 | 2 | 9 |
| *Pochonia* | 4 | 2 | 2 | 5 |
| *Podospora* | 2 | 0 | 2 | 9 |
| *Pseudocercospora* | 2 | 1 | 1 | 6 |
| *Pseudozyma* | 3 | 2 | 1 | 4 |
| *Puccinia* | 8 | 1 | 7 | 9 |
| *Punctularia* | 4 | 3 | 1 | 4 |
| *Purpureocillium* | 1 | 0 | 1 | 4 |
| *Pyrenophora* | 1 | 1 | 0 | 8 |
| *Rasamsonia* | 2 | 2 | 0 | 6 |
| *Rhinocladiella* | 3 | 1 | 2 | 9 |
| *Rosellinia* | 2 | 2 | 0 | 3 |
| *Saitoella* | 1 | 0 | 1 | 3 |
| *Scedosporium* | 6 | 1 | 5 | 9 |
| *Schizophyllum* | 3 | 3 | 0 | 3 |
| *Schizosaccharomyces* | 9 | 1 | 8 | 9 |
| *Sclerotinia* | 2 | 2 | 0 | 8 |
| *Sordaria* | 3 | 0 | 3 | 9 |
| *Sphaerulina* | 5 | 0 | 5 | 8 |
| *Sporothrix* | 1 | 1 | 0 | 7 |
| *Stereum* | 4 | 0 | 4 | 7 |
| *Sugiyamaella* | 1 | 1 | 0 | 7 |
| *Talaromyces* | 1 | 1 | 0 | 9 |
| *Thielavia* | 1 | 0 | 1 | 9 |
| *Togninia* | 5 | 2 | 3 | 3 |
| *Torulaspora* | 31 | 31 | 0 | 6 |
| *Tremella* | 4 | 1 | 3 | 7 |
| *Trichophyton* | 5 | 0 | 5 | 9 |
| *Trichosporon* | 1 | 1 | 0 | 4 |
| *Tsuchiyaea* | 3 | 2 | 1 | 6 |
| *Tuber* | 3 | 1 | 2 | 8 |
| *Uncinocarpus* | 4 | 1 | 3 | 7 |
| *Ustilago* | 2 | 2 | 0 | 5 |
| *Vavraia* | 1 | 0 | 1 | 2 |
| *Verruconis* | 7 | 1 | 6 | 8 |
| *Verticilium* | 4 | 0 | 4 | 7 |
| *Xylona* | 9 | 1 | 8 | 8 |
| *Yarrowia* | 9 | 1 | 8 | 9 |
| *Zygosaccharomyces* | 1 | 1 | 0 | 9 |
| *Zymoseptoria* | 1 | 0 | 1 | 4 |
