# Supplementary material for: Mycobiome changes in the vitreous of post fever retinitis patients
Source: PLoS One. 2020 Nov 19;15(11):e0242138. doi: 10.1371/journal.pone.0242138 (PMC7676714; doi:10.1371/journal.pone.0242138)
Supplement: S6 Table — (DOCX) [file pone.0242138.s008.docx]

S6 Table: Presence or absence of discriminative genera of the present study in other ocular studies.

| Genera | Ocular surface (Shivaji et al., 2019) | Keratitis (Prashanthi et al., 2019) | Endophthalmitis (Gandhi et al., 2019) |
| --- | --- | --- | --- |
| *Setosphaeria* | + | + | + |
| *Arthroderma* | - | - | - |
| *Isaria* | - | - | - |
| *Paracoccidioides* | - | - | - |
| *Sordaria* | - | - | - |
| *Nectria* | + | + | - |
| *Saccharomyces* | + | + | - |
| *Exserohilum* | - | - | + |
| *Conisporium* | - | - | - |
| *Nematocida* | - | - | - |
| *Enterocytozoon* | - | - | - |
| *Microsporum* | - | - | - |
| *Lachancea* | - | - | - |
| *Paraphaeosphaeria* | - | - | - |
| *Trichoderma* | + | + | + |
| *Pseudogymnoascus* | - | - | - |
| *Kluyveromyces* | + | + | - |
